# Transition from temporary to durable right ventricular support after left ventricular assist device implantation

**DOI:** 10.1093/icvts/ivad004

**Published:** 2023-08-31

**Authors:** Pia Lanmueller, Christoph Starck, Evgenij Potapov, Joerg Kempfert

**Affiliations:** Department of Cardiothoracic and Vascular Surgery, Deutsches Herzzentrum der Charité, Berlin, Germany; DZHK (German Centre for Cardiovascular Research), Partner Site, Berlin, Germany; Department of Cardiothoracic and Vascular Surgery, Deutsches Herzzentrum der Charité, Berlin, Germany; DZHK (German Centre for Cardiovascular Research), Partner Site, Berlin, Germany; Steinbeis University Berlin, Institute (STI) of Cardiovascular Perfusion, Berlin, Germany; Department of Cardiothoracic and Vascular Surgery, Deutsches Herzzentrum der Charité, Berlin, Germany; DZHK (German Centre for Cardiovascular Research), Partner Site, Berlin, Germany; Department of Cardiothoracic and Vascular Surgery, Deutsches Herzzentrum der Charité, Berlin, Germany; DZHK (German Centre for Cardiovascular Research), Partner Site, Berlin, Germany

**Conflict of interest:** Evgenij Potapov is a proctor and consultant for Abbott and Abiomed. Christoph Starck declares payment to his institution related to his activity as speaker and consultant for Abiomed. Pia Lanmueller and Joerg Kempfert have nothing to disclose.

## Reviewer information

Interdisciplinary CardioVascular and Thoracic Surgery thanks Carlos A. Mestres, Amber Malhotra and the other, anonymous reviewer(s) for their contribution to the peer review process of this article.

## Notes

Presented on October 6th at the 36th EACTS Annual Meeting, Milan, Italy.

